# Functional assessment of stretch hyperreflexia in children with cerebral palsy using treadmill perturbations

**DOI:** 10.1186/s12984-021-00940-1

**Published:** 2021-10-18

**Authors:** Eline Flux, Marjolein M. van der Krogt, Jaap Harlaar, Annemieke I. Buizer, Lizeth H. Sloot

**Affiliations:** 1 Department of Rehabilitation Medicine, Amsterdam Movement Sciences, Amsterdam UMC, Vrije Universiteit Amsterdam, Boelelaan 1117, PO Box 7057, 1007MB Amsterdam, The Netherlands; 2 Department Biomechanical Engineering, Delft University of Technology, Delft, The Netherlands; 3 Department Orthopedics, Erasmus Medical Center, Rotterdam, The Netherlands; 4 Emma Children’s Hospital, Amsterdam UMC, University of Amsterdam, Vrije Universiteit, Amsterdam, The Netherlands; 5 Institute for Computer Engineering, Heidelberg University, Heidelberg, Germany

**Keywords:** Spasticity, Stretch hyperreflexia, Perturbations, Cerebral palsy, Gait

## Abstract

**Background:**

As hyperactive muscle stretch reflexes hinder movement in patients with central nervous system disorders, they are a common target of treatment. To improve treatment evaluation, hyperactive reflexes should be assessed during activities as walking rather than passively. This study systematically explores the feasibility, reliability and validity of sudden treadmill perturbations to evoke and quantify calf muscle stretch reflexes during walking in children with neurological disorders.

**Methods:**

We performed an observational cross-sectional study including 24 children with cerebral palsy (CP; 6–16 years) and 14 typically developing children (TD; 6–15 years). Short belt accelerations were applied at three different intensities while children walked at comfortable speed. Lower leg kinematics, musculo-tendon lengthening and velocity, muscle activity and spatiotemporal parameters were measured to analyze perturbation responses.

**Results:**

We first demonstrated protocol feasibility: the protocol was completed by all but three children who ceased participation due to fatigue. All remaining children were able to maintain their gait pattern during perturbation trials without anticipatory adaptations in ankle kinematics, spatiotemporal parameters and muscle activity. Second, we showed the protocol’s reliability: there was no systematic change in muscle response over time (P = 0.21–0.54) and a bootstrapping procedure indicated sufficient number of perturbations, as the last perturbation repetition only reduced variability by ~ 2%. Third, we evaluated construct validity by showing that responses comply with neurophysiological criteria for stretch reflexes: perturbations superimposed calf muscle lengthening (P < 0.001 for both CP and TD) in all but one participant. This elicited increased calf muscle activity (359 ± 190% for CP and 231 ± 68% for TD, both P < 0.001) in the gastrocnemius medialis muscle, which increased with perturbation intensity (P < 0.001), according to the velocity-dependent nature of stretch reflexes. Finally, construct validity was shown from a clinical perspective: stretch reflexes were 1.7 times higher for CP than TD for the gastrocnemius medialis muscle (P = 0.017).

**Conclusions:**

The feasibility and reliability of the protocol, as well as the construct validity—shown by the exaggerated velocity-dependent nature of the measured responses—strongly support the use of treadmill perturbations to quantify stretch hyperreflexia during gait. We therefore provided a framework which can be used to inform clinical decision making and treatment evaluation.

**Supplementary Information:**

The online version contains supplementary material available at 10.1186/s12984-021-00940-1.

## Background

Stretch hyperreflexia, also known as spasticity, is considered one of the key impairments in upper motor neuron syndromes such as cerebral palsy, which is the main cause of childhood-onset disability [1]. Stretch hyperreflexia is commonly defined as exaggerated velocity dependent stretch reflexes [2] and likely caused by supraspinal disinhibition of the stretch reflex loop [3]. These overactive reflexes cause muscle contractions that often limit lengthening of muscles, leading to significant restrictions in motion of the joints. The abnormal muscle activity patterns affect daily life activities, such as walking [4]. Stretch hyperreflexia is often associated with other neural and non-neural impairments, such as increased background muscle activation (neural) and altered tissue properties (non-neural) [5]. Such tissue alterations include stiffer extracellular matrices, due to for instance increased collagen, which can lead to impaired muscle length and bony deformities [6–8]. The type of impairment guides the appropriate treatment. Neural impairments, including stretch hyperreflexia, can be treated with botulinum toxin injections, oral or intrathecal baclofen, or selective dorsal rhizotomy [9–11], with exclusively the latter directly targeting stretch hyperreflexia by mechanically intervening the stretch-reflex loop. Non-neural impairments, on the other hand, are often treated with stretching, corrective casting, splinting or surgery to lengthen the muscle [10, 12]. To select the appropriate treatment, diagnostic tests need to correctly identify the underlying neuromuscular problems.

The current clinical standard to assess stretch hyperreflexia is to apply manual rotations around a relaxed joint in a passive patient, and rate the perceived resistance to fast stretch of the muscle–tendon complex according to one of several available clinical scales [13, 14]. Unfortunately, the subjective and qualitative nature of these tests does not allow for sufficient and reliable discrimination between underlying causes to inform treatment [2, 15, 16]. One could for example easily misinterpret increased passive tissue stiffness as neurological driven stiffness, or vice versa, as both present with increased resistance to stretch. Furthermore, assessment of stretch hyperreflexia in relaxed limbs for interpretation in activities like walking is criticized [17, 18], as the magnitude of reflexes is known to be centrally regulated based on the activity. For instance, reflex magnitudes decrease from sitting to standing [19] and further decrease when walking [20, 21] and even adapt to the phase of the gait cycle [4, 22, 23]. Therefore, stretch hyperreflexia is best directly assessed during activities such as walking.

Several approaches have been suggested to assess reflexes during gait [24–29]. An actuated ankle orthosis for example has been shown to stretch and thus evoke calf muscle stretch reflexes during the stance phase of gait [22, 25, 30], but it is unknown to what extent the mass and movement restrictions of such an orthosis alter gait. Similar to the ankle orthosis, treadmill perturbations can be applied to evoke (hyper) reflexes in the lower leg muscles during walking [26, 29, 31]. In these perturbation methods, the running treadmill belt under the standing foot is momentarily decelerated [26, 29] or accelerated [26, 31], shortly increasing ankle plantar- or dorsiflexion and thus stretching the lower leg muscles, and evoking stretch reflexes. Up to now, treadmill perturbations have only been studied in able-bodied adults [26, 29, 31]. The next step is to evaluate this approach in patients with stretch hyperreflexia, such as children with spastic cerebral palsy. Children with cerebral palsy often present with abnormal gait patterns, and the altered ankle kinematics and lower gait stability [32] could interfere with the feasibility of the protocol and the effectiveness of evoking reflexes.

Therefore, the aim of our study is to analyze whether the treadmill perturbation protocol can evoke and quantify calf muscle stretch reflexes in children with cerebral palsy and in typically developing controls. We focus on the calf muscles, as they are the largest contributor to impaired gait in most children with cerebral palsy [33]. This paper is composed of three aspects: (1) protocol feasibility; whether we can apply perturbations while children maintain their walking pattern, (2) reliability; whether repeated perturbations present similar results, and (3) construct validity; whether the evoked muscle responses comply with the neurophysiological characteristics of stretch reflexes [26], and whether patients with spastic cerebral palsy can be distinguished from controls. We hypothesized that the perturbation protocol is feasible in all children, presents sufficient reliability and indeed evokes reflex activity. Furthermore, we anticipated larger stretch reflexes in children with cerebral palsy versus typically developing children.

## Methods

### Participants

Twenty-four children with spastic cerebral palsy and fourteen typically developing children participated (see Table 1) in this observational cross-sectional study. Inclusion criteria were: aged between 5 and 16 years, able to follow simple instructions and walk for approximately half an hour in total with sufficient rest. Specific inclusion criteria for the cerebral palsy group were: a diagnosis of uni- or bilateral spastic cerebral palsy with gross motor function classification system [34] level I-II. Children were excluded if they had recently received treatment that consisted of functional surgery on the legs or lower limb botulinum toxin-A injections in the past 6 months, had visual deficits, frequent epilepsy, behavioral problems or comorbidities that affect walking. Children who had undergone selective dorsal rhizotomy (SDR) were included if the recovery period of 12 months was satisfied. These children were analyzed separately in the clinical applicability section, due to the severe impact of the SDR surgery on reflex sizes. Passive levels of spasticity were determined using the SPAT [13], by stretching the calf muscles at slow and fast velocities, grading the intensity of muscle resistance during the fast velocity on a 0–3 scale. The SPAT score could not be determined in the case of clonus. Specific exclusion criteria for the typically developing group consisted of a history of neurological or orthopedic diseases.

**Table 1:** Participant characteristics

	GMFCS I/II	Distribution Uni/bi	Gender F/M	Age (y)	Height (m)	Weight (kg)	SPAT GM 0/I/II/III/CL	SPAT SO 0/I/II/III/CL	CWS (m/s)
TD	–	–	7/7	10.7 ± 3.2	1.50 ± 0.19	40.3 ± 13.0			1.19 ± 0.15
CP	9/9	4/14	6/12	10.1 ± 3.1	1.49 ± 0.19	37.2 ± 14.2	7/1/-/2/8	8/1/-/3/8	0.67 ± 0.19
SDR	0/3	0/3	2/1	11.3 ± 3.5	1.51 ± 0.16	43.0 ± 18.8	3/-/-/-/-	3/-/-/-/-	0.62 ± 0.24

Participant characteristics were included from all children who completed the protocol. *GMFCS* gross motor function classification system, [34] *GM* gastrocnemius medialis muscle, *SO* soleus muscle, scores reflect values of spasticity according to the SPAT test, [13] *Cl* clonus, no SPAT can be quantified for these participants, *CWS* comfortable walking speed on the treadmill, *TD* typically developing, *CP* cerebral palsy, *SDR* selective dorsal rhizotomy, children who had SDR surgery were excluded from analyses of neurophysiological criteria

### Protocol

**Fig. 1:**
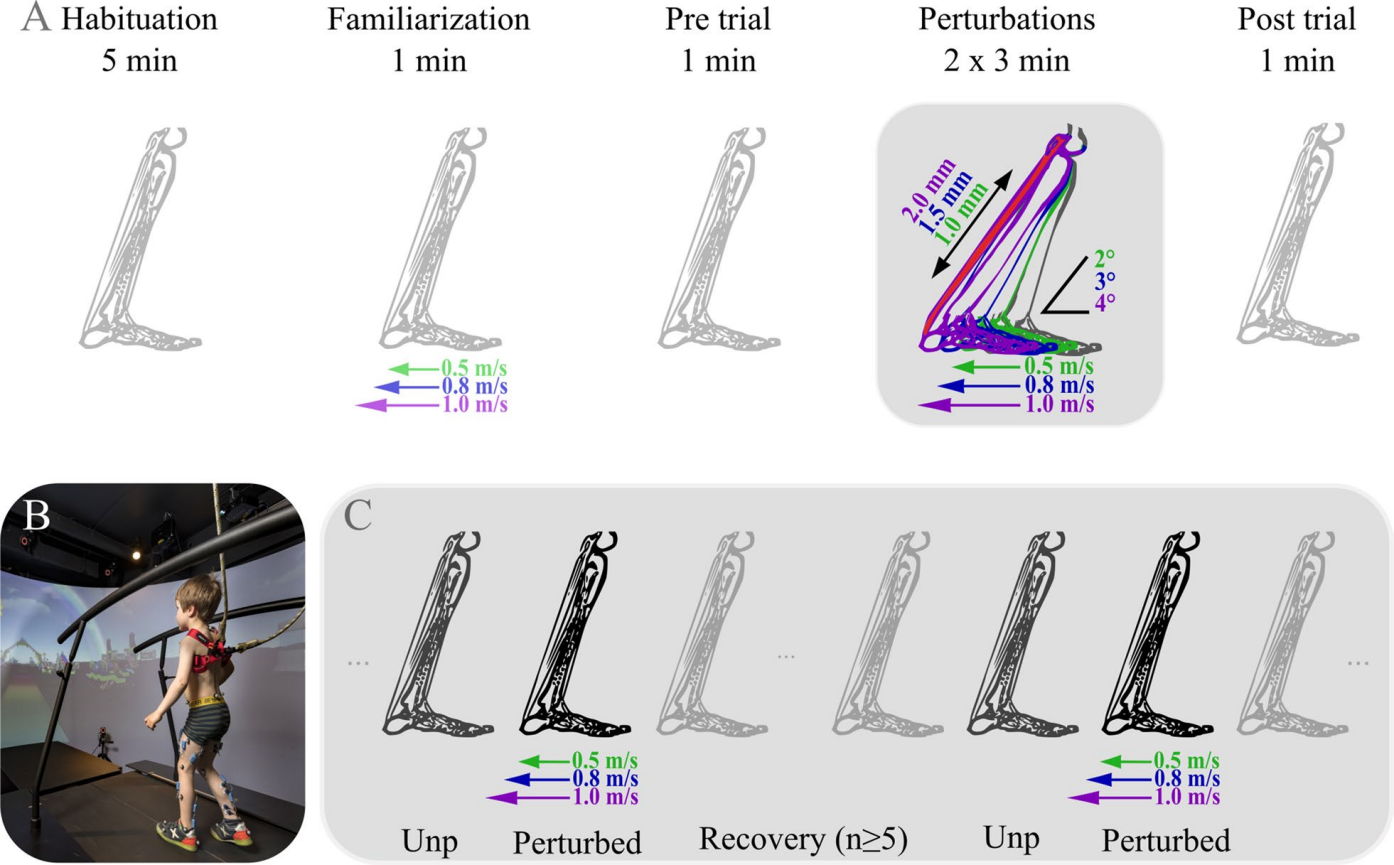
Perturbation protocol. **A** Schematic representation of the perturbation protocol. Perturbations of three different intensities were applied over two perturbation trials. **B** Measurement setup. **C** Schematic representation of the perturbation trial: At least five recovery strides occurred between perturbations. Each step before the perturbed trial was used for the average unperturbed (Unp) baseline measures

Participants walked on a split-belt instrumented treadmill in a virtual reality environment (Fig. 1, GRAIL, Motek ForceLink BV, Amsterdam, Netherlands) following the protocol as described by Sloot et al. [26]. Participants wore their own flat shoes and were secured by a non-weight-bearing safety harness attached to the ceiling. Ground reaction forces were measured at 1000 Hz by force sensors mounted underneath both treadmill belts (50 × 200 cm; R-Mill, Forcelink, The Netherlands). Motion data was captured at 100 Hz using a motion capture system (Vicon Motion Systems, Oxford, UK) and the Human Body Model marker set [35, 36]. EMG electrodes (bipolar, Ø 15 mm, 24 mm inter-electrode distance) were placed on the gastrocnemius medialis (GM), soleus (SO) and tibialis anterior (TA) muscle bellies of both legs according to SENIAM guidelines [37]. EMG was measured at 1000 Hz via a wireless system (Wave EMG system, Cometa, Italy).

Comfortable walking speed (Table 1) was determined at the start of the session for each participant individually by gradually increasing the belt speed until comfortable as indicated by child and parents. Participants walked for at least six minutes to habituate to the set-up [38, 39]. To familiarize to the protocol, participants received three perturbations of each intensity during the last habituation minute.

**Fig. 2:**
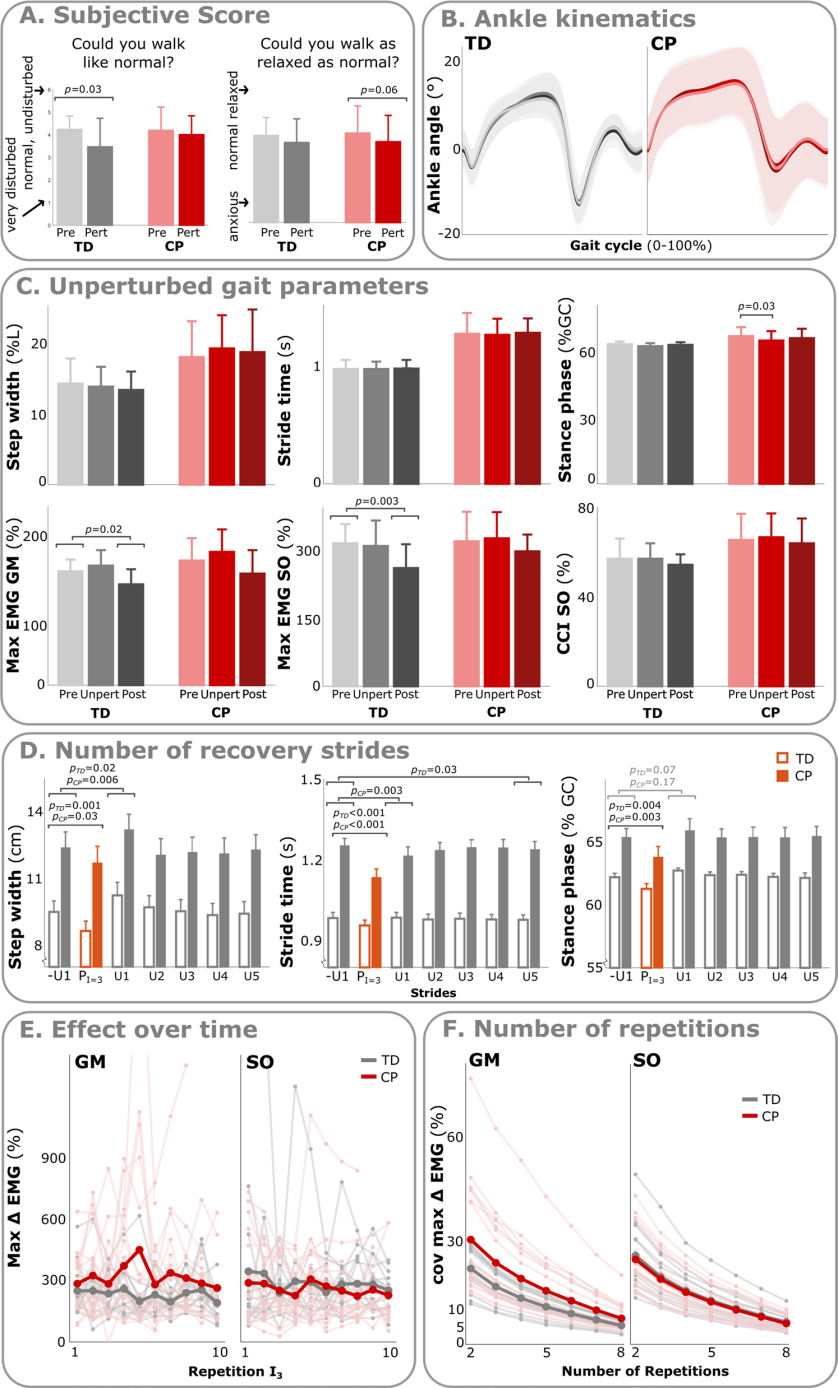
The feasibility (**A**–**D**) and reliability (**E**, **F**) of the perturbation protocol. p-values are indicated for the different variables if significant differences were found. Bar graphs indicate mean and standard deviation values. **A** Subjective rating of how relaxed and disturbed children with cerebral palsy (CP) and typically developing children (TD) felt before and after the perturbation trials. **B** Stride-cycle normalized ankle angle for both typically developing and cerebral palsy group during Pre and Post trials and unperturbed (unp)strides during perturbed trial. Color coding corresponds with that as indicated in the next sub-figure. **C** Comparison of parameters calculated for the Pre, Post and unperturbed strides for both participant groups. **D** Comparison between the unperturbed and perturbed strides and the five recovery strides for both groups using t-tests (group indicated at the specific p-value). **E** Evaluation of the GM and SO muscle response to the highest intensity perturbation over time (i.e., repetitions) for both groups in bold as well as the individual values in lighter colors. EMG is normalized to the average maximum of each participant during push-off of the unperturbed strides. Note that the perturbations were applied during two different trials with a short break in between, which did not visibly affect the results. **F** Coefficient of variation (COV) between the 1000 bootstrap repetitions per subset of n (2–8) random selection of strides from the available perturbed strides per participant and the average over participants in bold

Measurements started with a trial of one minute of unperturbed walking (Pre; Fig. 1), during which the absence of perturbations was explicitly mentioned to prevent cautious gait. Next, perturbations were applied to the leg with the most spastic calf muscles (cerebral palsy) or the right leg (typically developing). Perturbations were applied over two trials of three minutes each, with a short break in between to limit fatigue. The protocol ended with another explicit unperturbed walking trial of one minute (Post). All participants reported that they were able to feel at least the two most intense perturbations. Therefore, we assessed if anticipation of perturbations influenced their gait pattern, by asking two questions on subjective walking experience (see Fig. 2A) after the Pre and after the perturbation trials.

**Fig. 3:**
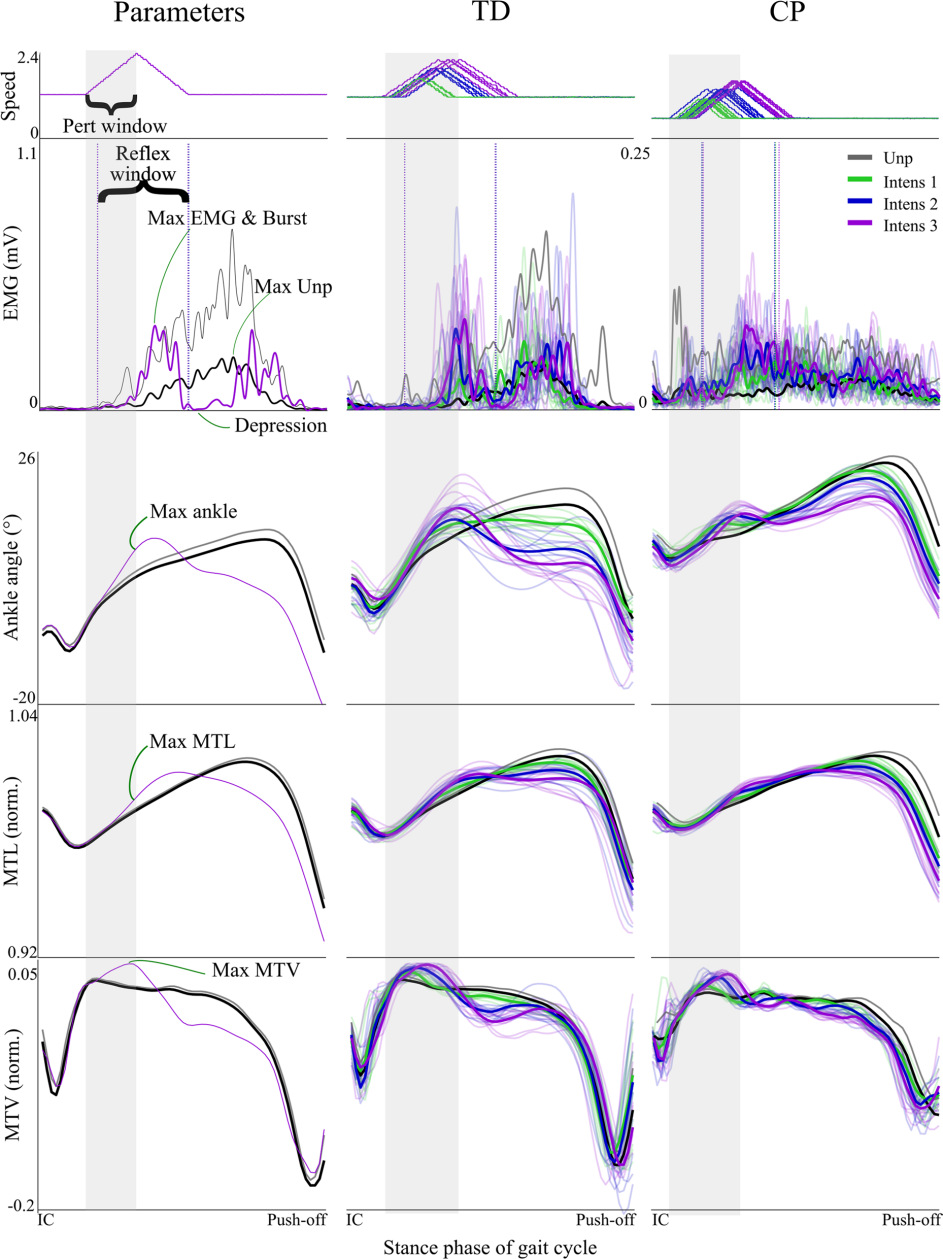
Muscle parameters and typical examples. Treadmill belt perturbations (speed) and the responses for the gastrocnemius medialis muscle are visualized for the different parameters (column 1) and for a typically developing child (TD; column 2) and a child with cerebral palsy (CP; column 3). Values are plotted from initial contact (IC) to push-off. Black represents average unperturbed walking, with grey indicating n × standard error (5 × for EMG and 1 × for ankle angles, MTL and MTV). Light shaded lines represent individual perturbed strides and bold darker colored lines represent average of perturbed strides for the three different intensities. *MTL* muscle tendon lengthening, *MTV* muscle tendon velocity, *mV* millivolt, *norm* normalized, *Unp* unperturbed

The treadmill perturbations consisted of a short acceleration of the belt during the first period of stance (Figs. 1, 3, Table 2), pulling the foot backwards and stretching the calf muscles. Three perturbation intensities were applied to evaluate how muscle activity response scales with muscle lengthening velocity. The intensities corresponded to an increased treadmill speed of 0.5 ms^−1^, 0.8 ms^−1^ and 1.0 ms^−1^. During each perturbation trial, four to six repetitions of each perturbation intensity were applied to random strides. The number of recovery strides was randomized but at least five strides before the next perturbation were applied. If feet stepped on both treadmill belts, no perturbations were applied to prevent falls.

**Table 2:** Perturbation characteristics

	I1	I2	I3	ANOVA	Posthoc
Peak Δ v (ms^−1^)	0.53 ± 0.01	0.80 ± 0.01	1.03 ± 0.01	**< 0.001**	All
Peak Δ vrel (%)	74.49 ± 31.32	111.60 ± 47.31	144.15 ± 60.99	**< 0.001**	All
Peak Δ a (ms^−2^)	18.08 ± 1.01	18.41 ± 1.37	18.34 ± 1.30	0.505	All
Time to peak v (ms)	72.67 ± 11.04	92.81 ± 15.32	103.75 ± 15.34	**< 0.001**	All
Time to peak vrel (%)	10.32 ± 0.75	13.24 ± 0.88	15.56 ± 1.04	**< 0.001**	All
Duration (ms)	143.71 ± 22.07	181.27 ± 29.40	206.69 ± 30.60	**< 0.001**	All
Duration rel (%)	20.37 ± 1.44	25.93 ± 1.78	30.95 ± 2.03	**< 0.001**	All
Start (ms)	95.81 ± 51.93	80.34 ± 18.40	77.92 ± 18.63	0.063	–
Start rel (%)	11.64 ± 2.35	11.88 ± 2.72	11.94 ± 2.58	0.877	–
Start rel TD (%)	12.02 ± 1.48	12.11 ± 1.57	11.88 ± 1.53	0.661	–
Start rel CP (%)	11.38 ± 3.61	11.73 ± 3.98	11.99 ± 3.32	0.626	–
Peak Δ Ankle angle (°)	1.99 ± 0.69	3.60 ± 0.94	4.89 ± 1.49	**< 0.001**	All
Peak Δ Ankle ω (°s^−1^)	45.65 ± 14.65	62.21 ± 16.61	77.83 ± 24.00	**< 0.001**	All

Spatiotemporal parameters (Time to peak v, perturbation duration and perturbation start) are expressed in absolute values as well as relative to the gait cycle (rel). Mean ± standard deviations are presented. Δ v = difference in treadmill velocity, Δvrel = difference in treadmill velocity relative to participant’s belt velocity, a = treadmill acceleration, ω = angular velocity. Significant values are expressed in bold. All posthoc testing revealed significant differences between all conditions (p < 0.05)

### Data processing

3D joint angles for the hip, knee and ankle, as well as musculo-tendon lengths (MTL) of the Gastrocnemius Medialis (GM), Soleus (SO) and Tibialis Anterior (TA) muscles were calculated using the generic gait model (GAIT2392) in musculoskeletal modeling software (OpenSim) [40]. In this software, the model was scaled to fit the individual participants and the inverse kinematics tool was used to fit the individual participant’s kinematics [26]. Further calculations were performed using custom made code in Matlab (The Mathworks Inc., Natick MA; version 2019a). MTL was differentiated to obtain musculo-tendon stretch velocity (MTV). Kinematics, MTL and MTV were low-pass filtered (bi-directional 4th order Butterworth at 20 Hz) and non-dimensionalized by dividing MTL by the anatomical reference length with all joint angles set at zero (l_ref_) and MTV by √(g∙l_ref_) [27, 41].

EMG signals were high-pass filtered (bidirectional 4^th^ order Butterworth at 20 Hz), rectified and low-pass filtered (at 50 Hz). All EMG signals were normalized to the peak during each muscles’ region of interest—defined as swing for TA and push-off for GM and SO—obtained from the further smoothed (8 Hz low-pass) Pre trial signals. Push-off was defined as the moment of zero crossing of anterior–posterior force to toe-off. Initial foot contact and toe-off values were determined using the horizontal position of the heel, toe and pelvic markers, conform the method of Zeni et al. [42], both online to predict initial contact for treadmill perturbation onset, as well as offline to determine actual initial contact. The latter were used to time-normalize belt speed, kinematics, MTL, MTV and EMG signals.

### Data analysis and statistics

- Protocol feasibility
  As part of the protocol feasibility, we evaluated any anticipatory changes in children’s gait pattern; if the number of steps in between perturbations was sufficient to return to normal after a perturbation; and if timing of the perturbations was consistent. All feasibility analyses were performed for the cerebral palsy and typically developing group separately.
  We first evaluated how many children could complete the entire protocol and reported any adverse events such as falls, failures or discomfort. Additionally, we evaluated whether children were able to maintain their walking pattern despite perturbations. We did this by comparing the subjective rating of their gait before and after the perturbation trial (Fig. 2A) using Wilcoxon Signed Ranks Test. Likewise, we quantitatively examined any changes in walking pattern by comparing the unperturbed strides directly preceding the perturbed strides with walking when no perturbations were anticipated, i.e., the last ten strides during both Pre and Post trials. Both Pre and Post were included to discriminate between fatigue and perturbation effects [43]. One cerebral palsy and one typically developing child were excluded from this sub-analysis due to missing data for the Post trial. We compared several outcome parameters that are known to be affected by anxiety; i.e., we checked for shorter step length and duration, longer stance phase, smaller step width, increased knee and ankle flexion, as well as reduced peak muscle activation in the GM and SO or increased co-contraction of these muscles with the TA [43]. Co-contraction was measured using the co-contraction index according to the following equation:
  1 $$co-contraction\, index\,=\,1- \frac{|{EMG}_{ag}- {EMG}_{ant}|}{{EMG}_{ag}+ {EMG}_{ant}}$$
  with EMG_ag_ the agonist (GM and SO) and EMG_ant_ the antagonist muscle (TA), with 1 full and 0 absence of co-contraction [44]. A repeated measures ANOVA was performed to compare the Pre, unperturbed and Post strides, and in case of significance post hoc paired sample t-tests were performed.
- Protocol reliability
  For reliability of our protocol we first assessed if the number of recovery strides was sufficient to return to unperturbed walking, by comparing step width, stride time and stance phase between unperturbed versus perturbed and the five recovery strides using paired t-tests without correction for multiple comparison as to not underestimate any differences [26]. We also evaluated the timing of the perturbations, as the more variable gait pattern of children with cerebral palsy [45] might affect the online predicted initial foot contact and thus the repeatability of perturbations. We reported the perturbation onsets and compared the standard deviation from the cerebral palsy group with the typically developing group (one-tailed independent samples t-test). We furthermore analyzed if perturbation velocity, acceleration and duration and resulting ankle dorsiflexion increased with intensity, using repeated measures ANOVAs with linear polynomial contrast and post hoc independent t-tests.
  Next, the within session reliability of the perturbation protocol was assessed as the consistency of response to repeated perturbations, to evaluate if there was habituation to the perturbations reducing the effectiveness of the protocol. We assessed if we could reliably estimate increases in GM and SO EMG (as defined in the validity section) for the highest intensity perturbations. Changes in muscle response size over time were evaluated for both muscles using a repeated measure ANOVA with a polynomial contrast per participant group. We furthermore assessed the required number of repetitions using a bootstrap procedure: for every participant, randomly selected perturbation strides were drawn from the available perturbations and this was repeated 1.000 times. The coefficient of variation, defined as the standard deviation over the 1.000 samples normalized to the mean, was established for every number of perturbations (ranging from 2 to 8 perturbations) and averaged over participants. When the coefficient of variation values reached a plateau, it was assumed that sufficient numbers of repetitions were included.
- Construct validity of stretch reflexes
  To assess the construct validity of the perturbation protocol, we first evaluated evoked muscle responses against three neurophysiological criteria, similar to our previous work [26]. All neurophysiological criteria are based on the commonly presumed velocity dependent character of stretch reflexes [2]:
  1. Mechanical response: increasing perturbation intensity should result in an increase in MTL and MTV;
  2. Electrophysiological response: increasing perturbation intensity should evoke an increasing burst of muscle activity in the stretched muscle;
  3. The burst in muscle activity should not (solely) be related to co-contraction with the antagonist muscle.

To evaluate criterium 1, the peak values of the ankle and knee angles as well as GM and SO MTL and MTV were calculated by subtracting each individual perturbed stride from unperturbed walking, that is the average of all strides directly before a perturbed stride (see Fig. 1). These values are further referred to as Δankle dorsiflexion, Δknee flexion, ΔMTL and ΔMTV. We additionally analyzed the percentage of trials with a successful mechanical response, defined as a ΔMTV above one standard deviation of unperturbed MTV. Participants without a mechanical response were reported, and neglected for further analysis.

Criterium 2, the GM and SO electrophysiological response (see Fig. 3), was calculated as the maximum difference in EMG (ΔEMG) between individual perturbed and average unperturbed strides during the reflex time window. This window was set as 35 ms after start of the perturbation until 120 ms after the maximum of the perturbation, thus based on the minimal and longest expected stretch reflex delay in calf muscles [17]. Furthermore, we analyzed the percentage of trials with occurrence of a burst. According to our previous work [26], a burst was defined as an increase in EMG activity exceeding five times the standard deviation of unperturbed EMG activity for at least 8 ms during the reflex window (Fig. 3). To evaluate criterium 3, the amount of co-contraction was calculated within the reflex time window using Eq. 1.

Furthermore, we assessed the construct validity of the perturbation protocol from a clinical perspective. This was performed by assessing whether the protocol could distinguish children with cerebral palsy from typically developing children. We furthermore contrasted this with preliminary outcomes of three children who underwent SDR. We calculated the muscular response strength, defined as ΔEMG divided by ΔMTV [26]. As children with cerebral palsy often have decreased post-activation depression or a continued increased activation [46], we also examined the duration of the muscular response. GM and SO ΔEMG were averaged per 20 ms bins starting from perturbation onset to the end of stance. Response duration was defined as the longest continuous period of positive bins during stance.

### Statistical analysis

Numerical data was tested for normality before performing parametric testing. All statistical tests were two-tailed and P < 0.05 was considered statistically significant, unless indicated otherwise, and analyses were performed in IBM SPSS Statistics version 26 (Armonk, NY, USA). Statistical analyses for feasibility and reliability measures are described within their sections. Neurophysiological criteria for construct validity were statistically analyzed using a repeated measures ANOVA with the three criteria as dependent factors and cerebral palsy/typically developing as grouping factor. Children who underwent SDR surgery were excluded from this analysis, as no enhanced reflexes were expected in this group. Contrasts were used to examine whether the parameters differed between unperturbed and perturbed strides (Helmert contrast; P_pert_), whether this increased with perturbation intensity (linear polynomial contrast; P_intensity_), and in particular if this increase had an interaction effect with group (cerebral palsy/typically developing, using linear polynomial contrast; P_inter_). The intensity contrast was additionally assessed for cerebral palsy (P_CP_) and typically developing (P_TD_) separately. The clinical criteria, muscular response strength and duration, were compared between participant groups using a repeated measures ANOVA (linear polynomial contrast; P_group_). Due to the small sample size, SDR muscular response strength and duration were only visually compared.

## Results

### Protocol feasibility

All but three children with cerebral palsy finished the protocol, two of which (aged 9 and 12 years) terminated early due to fatigue complaints and one participant (aged 9 years) did not want to continue after the perturbation habituation trial without an explicit reason. No falls occurred and none of the other participants reported discomfort or pain. Two participants reported anxiety for the perturbations but finished the protocol nonetheless. Further analysis was continued with the 21 children with cerebral palsy that finished the protocol.

The gait of both participant groups was not considerably altered during the perturbation trials compared with walking during the Pre trial. Generally, the children rated their gait pattern as normal and relaxed (Fig. 2A), with typically developing feeling slightly more disturbed during the perturbation trial (*z* = −2.16, *P* = 0.03) but as relaxed as normal (*z* = −1.03, *P* = 0.31); while cerebral palsy did not feel more disturbed (*z* = −1.24, *P* = 0.22) but showed a trend of feeling slightly less relaxed (*z* = −1.85, *P* = 0.06). This aligns with the quantitative analysis of the walking pattern, and specifically the potential effect on spatiotemporal parameters, ankle and knee flexion and peak muscle activation. Ankle angles were very similar between Pre, Post and unperturbed strides (Fig. 2B). For typically developing, the only difference was a decrease in GM (*P*_ANOVA_ = 0.04) and SO (*P*_ANOVA_ = 0.003) muscle activation between Pre and Post trials (Fig. 2C), with no significant differences with the unperturbed strides (*P* = 0.19 with Pre and *P* = 0.20 with Post). In the cerebral palsy group, only stance phase duration was reduced by 2.0% (*P* = 0.03) during perturbed versus Pre trial (Fig. 2C).

### Protocol reliability

The number of recovery strides were sufficient for the children to return to normal gait, with no significant differences from the second recovery stride onwards (Fig. 2D). Perturbations started on average around 12 ± 3% of the gait cycle and were all applied during the stance phase of gait (Table 2). Onsets did not differ across the different intensities (*P* = 0.877), although the standard deviation of the onset was significantly higher for cerebral palsy (*P* = 0.028). Perturbation intensity increased with higher intensities (*P* < 0.001; Table 2).

The muscular response strength to the perturbations did not change systematically with repeated perturbations (Fig. 2E; cerebral palsy GM: *P* = 0.28, SO: *P* = 0.21; typically developing GM: *P* = 0.29, SO: *P* = 0.54). The lack of habituation to the perturbations allowed us to average over multiple repetitions to reduce the coefficient of variation. Including up to 8 repetitions reduced the coefficient of variation to below 7% for typically developing and 8% for cerebral palsy and almost reached a plateau (Fig. 2F) with a reduction of around 2% for the latest perturbation.

### Construct validity

**Fig. 4:**
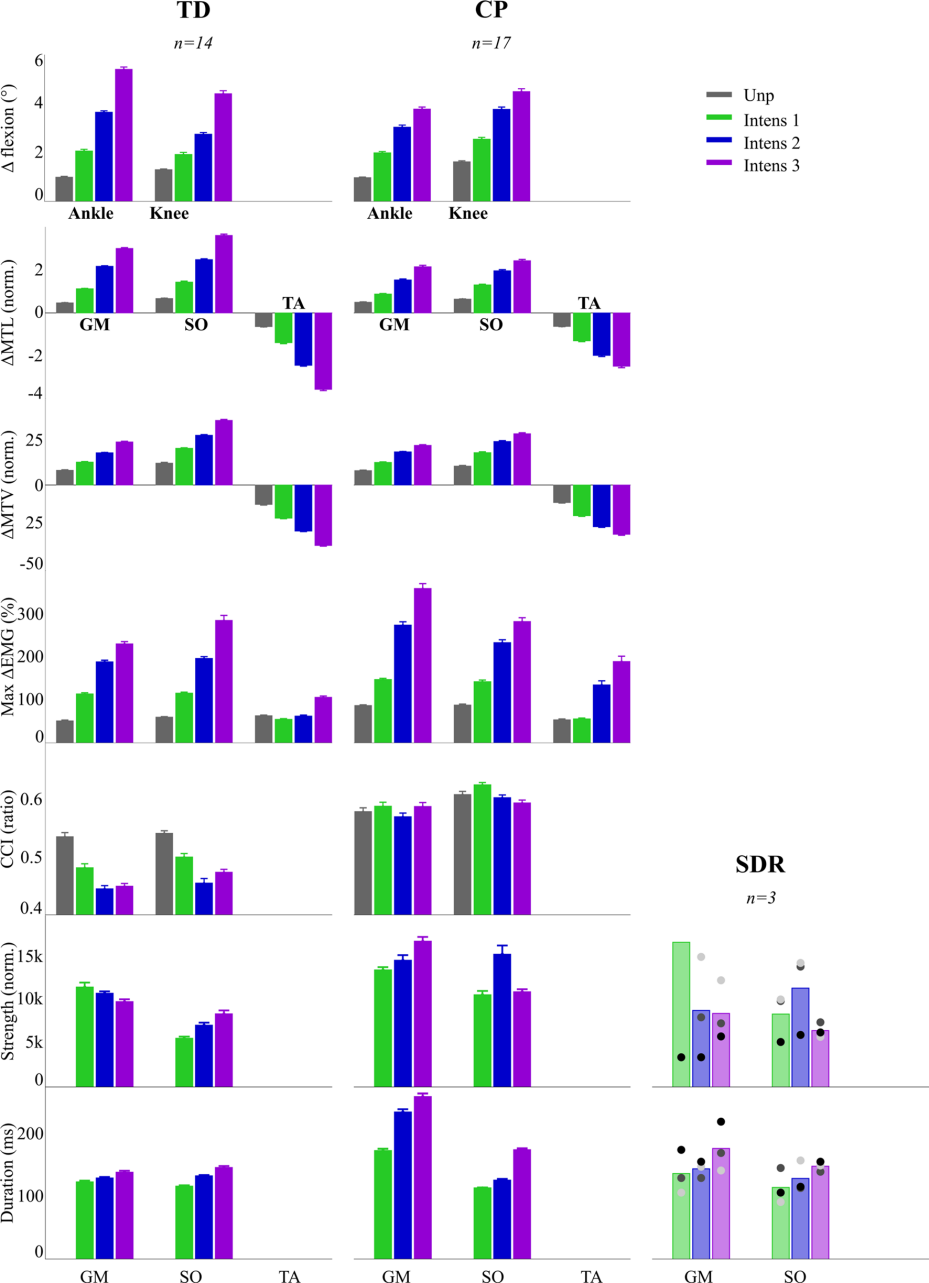
Construct validity of stretch reflexes: the three neurophysciological criteria for stretch reflexes are shown (row 1–5), as well as the clinical criteria (row 6, 7). (1) The mechanical response for the ankle and knee joint; and (2, 3) of the gastrocnemius medialis (GM), soleus (SO) and tibialis anterior (TA) muscle; (4) the electrophysiological response for the GM, SO and TA; (5) the amount of co-contraction; (6) muscular response strength; and (7) response duration. Bar graphs indicate mean and standard error of the mean values. Values are presented for unperturbed (unp) and perturbation intensity (Intens) 1–3. For the clinical criteria, only I1-3 are shown. Values for the selective dorsal rhizotomy (SDR) group are only presented for clinical criteria. *MTL* muscle tendon lengthening, *MTV* muscle tendon velocity, *CCI* co-contraction index; *norm* normalized

All children but one had successful mechanical responses to the perturbations (i.e., ΔMTV above one standard deviation of unperturbed MTV; Table 3) in at least the highest intensity, fulfilling criterium 1. The exception was one child with cerebral palsy that exhibited considerable reduced ankle range of motion throughout the gait cycle, and as this anatomical constraint prohibited evoking a stretch reflex, this participant was excluded from further analysis. In the other participants, ankle dorsiflexion increased with on average 5.4° ± 1.3 for typically developing and 3.8° ± 1.4 for cerebral palsy for the highest intensity perturbations (Additional file 1: Table S1). The increased ankle dorsiflexion resulted in increased GM and SO ΔMTL and ΔMTV (*P*_*intensity*_ < 0.001; Table 4; Figs. 3, 4). The mechanical response increased with increasing perturbation intensity for ankle dorsiflexion, MTL and MTV (*P*_*intensity*_ < 0.001; Table 4; Figs. 3, 4). This increase in response was stronger in typically developing children compared to children with cerebral palsy (*P*_*inter*_ = 0.01 – 0.038), with the exception of GM ΔMTV (*P*_*inter*_ = 0.178). Knee flexion also increased due to perturbations, both in cerebral palsy (*P*_*CP*_ = 0.002) and typically developing (*P*_*TD*_ = 0.001; Additional file 1: Table S1, Figs. 3, 4).

**Table 3:** Mechanical and electrophysiological responses

	Mechanical			Electrophysiological		
	Intensity 1 %	Intensity 2 %	Intensity 3 %	Intensity 1 %	Intensity 2 %	Intensity 3 %
TD						
GM	93.9	99.4	98.2	30.5	66.3	78.4
SO	83.9	96.5	98.8	55.3	91.4	97.9
CP						
GM	91.1	97.2	98.7	22.8	55.1	69.6
SO	80.3	92.6	98.1	50.5	84.7	96.2

Percentage of perturbations with a successful mechanical (increase in ΔMTV above one standard deviation of unperturbed MTV) and electrophysiological (increase in EMG activity above five times the standard deviation of unperturbed EMG) response for the three different intensities

**Table 4:** Statistical outcomes for the construct validity evaluation

Parameters	TD	CP	P_pert_	P_intensity_	P_group_	P_inter_
Max Δangle (°)						
Ankle	5.40 ± 1.34	3.79 ± 1.40	**< 0.001**	**< 0.001**	**0.028**	**0.002**
Knee	4.41 ± 1.64	4.50 ± 1.90	**< 0.001**	**< 0.001**	0.153	0.430
Max ΔMTL (norm.)						
GM	2.99 ± 0.52	2.15 ± 1.05	**< 0.001**	**< 0.001**	**0.006**	**0.038**
SO	3.60 ± 0.89	2.42 ± 1.02	**< 0.001**	**< 0.001**	**0.016**	**0.001**
TA	− 3.56 ± 0.81	− 2.49 ± 1.05	**< 0.001**	**< 0.001**	**0.028**	**0.002**
Max ΔMTV (norm.)						
GM	25.04 ± 4.04	23.05 ± 5.57	**< 0.001**	**< 0.001**	0.682	0.178
SO	37.51 ± 7.40	29.76 ± 9.78	**< 0.001**	**< 0.001**	0.057	**0.011**
TA	− 35.14 ± 5.36	− 28.72 ± 8.53	**< 0.001**	**< 0.001**	0.085	**0.006**
Max ΔEMG (%)						
GM	230.92 ± 67.91	359.53 ± 189.63	**< 0.001**	**< 0.001**	**0.018**	0.051
SO	285.10 ± 160.90	282.69 ± 149.86	**< 0.001**	**< 0.001**	0.540	0.537
TA	106.47 ± 39.83	189.88 ± 204.63	**0.003**	**0.001**	0.131	0.126
CCI difference						
GM	− 0.09 ± 0.06	0.01 ± 0.06	**< 0.001**	0.069	**0.001**	0.076
SO	− 0.07 ± 0.05	− 0.01 ± 0.05	**< 0.001**	**0.001**	**< 0.001**	0.742
Muscular response strength (norm.)						
GM	9.95 ± 3.74	16.96 ± 8.81	0.658	0.501	0.064	**0.045**
SO	8.52 ± 5.65	11.10 ± 5.05	0.521	0.237	**0.005**	0.351
Muscular response duration (ms)						
GM	102.95 ± 7.47	124.57 ± 15.39	**< 0.001**	**< 0.001**	**< 0.001**	**0.001**
SO	93.88 ± 8.65	114.07 ± 16.70	**< 0.001**	**< 0.001**	**0.004**	**0.001**

Values for the highest intensity are presented for all construct validity parameters. Values for unperturbed and the three intensities are presented in Additional file 1: Table S1. Abbreviations: with TD the typically developing group, CP the cerebral palsy group, P_pert_ the effect of perturbations (Helmert contrast), P_intensity_ the effect of intensities (linear polynomial contrast), P_group_ the difference between CP and TD, P_inter_ the interaction between CP and TD, *MTL* musculo-tendon length, *GM* gastrocnemius medialis muscle, *SO* soleus muscle, *TA* tibialis anterior muscle, *MTV* musculo-tendon stretch velocity, *CCI diff* difference in co-contraction index between unperturbed and highest intensity perturbations. Mean ± standard deviations are presented. Significant p-values are expressed in bold

The mechanical responses resulted in an electrophysiological response (i.e., a burst in EMG) in all children at least in the highest intensity (Table 3), fulfilling criterium 2. Likewise, increased muscle stretch of both muscles resulted in increased muscle activity (*P*_*intensity*_ < 0.001; Table 4; Figs. 3, 4). The electrophysiological response was overall higher for cerebral palsy than typically developing (*P*_*group*_ = 0.018) for the GM, but unlike the mechanical response, no interaction effect was present. For the SO no difference was found between cerebral palsy and typically developing (*P*_*group*_ = 0.540; *P*_*inter*_ = 0.537). Co-contraction did not increase due to perturbations, fulfilling criterium 3. In contrast, it decreased for the SO (*P*_*intensity*_ = 0.001), with a trend for GM (*P*_*intensity*_ = 0.069; Table 4; Figs. 3, 4), especially in typically developing children (*P*_*TD*_ = 0.008 for GM and *P*_*TD*_ = 0.006 for SO; see Additional file 1: Table S1).

As hypothesized, children with cerebral palsy not only had a higher muscular response strength (ΔEMG/ΔMTV), but the response also continued longer, thereby fulfilling the clinical criteria. The GM response was 48.7% higher (*P*_*group*_ = 0.017) and lasted 96.4% longer (*P*_*group*_ < 0.001) on average for cerebral palsy compared to typically developing (Table 4; Fig. 4). Similarly, the SO response showed a trend of 31.3% increase (*P*_*group*_ = 0.064), with large interindividual differences, and lasted 28% longer on average (*P*_*group*_ < 0.001) for cerebral palsy. The children who underwent SDR to reduce their reflex responses showed lower muscular response strength than children with cerebral palsy who did not underwent SDR surgery, despite similar mechanical responses.

## Discussion

This study is, to our knowledge, the first to investigate a perturbation protocol to evoke stretch reflexes in the calf muscles during gait in children with cerebral palsy and typically developing controls. The feasibility of the protocol was reflected by the low number of drop-outs and absence of noticeable gait adaptations in anticipation of the perturbations. We also showed an absence of habituation to the perturbations, allowing to reliably estimate the muscle response by averaging over repetitions. The construct validity of the protocol was based on the evoked muscle responses, showing muscle-lengthening velocity dependency conform the neurophysiological criteria of stretch reflexes. Furthermore, results complied with clinical criteria, as the protocol could distinguish between the group of children with spastic cerebral palsy and typically developing children.

### Protocol feasibility and reliability

Feasibility of the protocol in children as well as reliable responses are a prerequisite for clinical implementation. While three children did not finish the protocol, this was due to fatigue of this comprehensive protocol implementation and not the perturbations themselves. The children that did finish the protocol generally felt relaxed and although subjective scores showed that typically developing children were slightly more anxious during the perturbation trials, this was not reflected in their walking pattern. They did show significantly reduced maximum EMG between Pre and Post trials, but this was not visible in the perturbation trial and therefore more likely attributed to habituation or fatigue. Children with cerebral palsy did reduce their stance time during the perturbed trial, but this change was, if anything, not indicative of a more cautious walking pattern [47]. Furthermore, their perceived gait did not change due to the perturbations. There were no further indications for an adaptive gait pattern during the perturbation trials, hence we confirmed that the children maintained their gait pattern despite the perturbations. Overall, the protocol appears feasible, except maybe for children with severely reduced ankle range of motion, as it was not possible to evoke stretch reflexes in one such subject with the current perturbation settings.

Although the treadmill perturbations are designed to impose ankle dorsiflexion, any resulting knee flexion could interfere with stretching the bi-articular GM muscle. This can possibly explain differences between two previous studies applying perturbations of similar intensity around the ankle joint of healthy adults: Sinkjaer et al. [22] found short- and long-latency muscle responses to orthotic ankle perturbations, while Dietz et al. [30] only found long-latency muscle responses to perturbations applied using treadmill accelerations. Knee flexion angles were not reported in either study, but were likely increased in the latter study, as this was also the case in a similar study on healthy adults by Sloot et al. [26]. Even though we noted a large increase in knee flexion in our patients, we did elicit stretch in the GM muscle, resulting in reflex responses which even appeared higher than the SO muscle response. This is of particular interest because bi-articular muscles are more often targeted in stretch hyperreflexia treatment.

Our protocol distinguishes itself from other perturbation protocols [22, 25, 30] in that one uniform device is needed for all participants. Our perturbations require a treadmill with possibility for real-time perturbations, which are becoming increasingly popular in gait labs that treat more severely impaired patients with motor disorders, also driven by the increased evidence for perturbation-based training [48–53]. Furthermore, perturbation treadmill requirements are lower compared to those for previous treadmill perturbation studies [29, 31] with relatively low perturbation intensities (e.g., increased belt speed of 1 m/s compared to 6 m/s [31]).We previously applied perturbations of even lower intensity in able-bodied adults (increased belt speed of 0.5 m/s [26]), but shortened the duration of the perturbations in this protocol, as children have shorter stance duration. This resulted in more ankle flexion (2 and 1.3 times as large as in able-bodied adults [26] for typically developing and cerebral palsy respectively), but mechanical responses remained smaller compared to the treadmill perturbations from Berger et al. [31] (78°/s versus 250–300°/s ankle angular velocity, respectively) and orthotic perturbations [54] (5° versus 8° ankle dorsiflexion, respectively). Such more intense perturbations are needed to further elucidate the exact character of the muscle response, for instance to distinguish between short and long latency stretch reflexes [31, 54]. Besides the fact that such distinctions are difficult, even with more intense perturbations [55], these high intensity perturbations will be challenging in children with cerebral palsy and might cause instability. Furthermore, although a distinction is very interesting from a research perspective, this is not necessarily required for clinical purposes, nor assessed in current clinical stretch hyperreflexia measures. Given that our current velocities appear high enough to elicit muscle responses, we therefore recommend similar perturbations for clinical implementation.

The feasibility can be further improved through shortening the protocol, by removing baseline assessments and reducing the number of recovery strides, as children were generally stable after the second recovery stride. Furthermore, habituation might not be necessary for the stretch hyperreflexia assessment, as long as people feel comfortable, especially for participants already familiar with treadmill walking. Additionally, repetitions could be reduced as we showed stable responses and acceptable coefficient of variation after eight repetitions. Thus, we recommend a perturbation protocol of similar intensities with eight repetitions and three recovery strides for implementation. Future research could focus on the need for three different intensities or alternatively a range of intensities that can be online adjusted based on the mechanical response.

### Validity of stretch reflexes

The type of evoked muscle response, and whether or not these are due to stretch reflexes, is important to establish. Stretch reflexes are generally accepted to be velocity dependent. Clinical tests utilize this dependency by comparing perceived resistance between fast and slowly applied rotations around joints to discriminate between presumed stretch reflexes and other effects. However, part of the velocity dependent resistance can be caused by the viscoelastic component of the muscle–tendon complex [56], therefore stretch hyperreflexia measures should include muscle responses to discriminate between passive and reflexive resistance. The muscle responses to our perturbations clearly increased with increasing perturbation intensity and hence with musculo-tendon lengthening velocity, without being caused by increasing co-contraction as part of for instance a stabilization strategy. Therefore, responses comply with neurophysiological criteria for reflexes. However, we assessed muscle activity in a relatively long response time window (190–220 ms), based on the perturbation duration (70–100 ms to maximum velocity), to ensure that all reflexive activity was included. Given the long window, we cannot rule out that responses are partly caused by voluntary activity and other trans-cortical contributions [55]. Nevertheless, two findings from a clinical perspective further support the reflexive nature of the responses. First, responses were higher in children with cerebral palsy, who are known to have increased reflexes [57] and decreased voluntary activity [58]. In line with this are the low muscular responses found in the SDR group, who are expected to have considerably decreased reflexes. Secondly, calf muscles remained increasingly active for a longer duration in children with cerebral palsy. This finding can be explained by decreased post-activation depression, which has been related to stretch hyperreflexia [46, 59]. Accordingly, visual inspection of the data showed that only some of the children with cerebral palsy (4/23) showed a clear depression after peak activity, whereas this was visible in most typically developing children (10/14). All findings combined suggest that the protocol indeed appeared to evoke stretch reflex activity.

To be useful in the clinic, the perturbation protocol should be able to identify abnormal stretch reflex activity in patients. In this study, we established the first step: the protocol was able to discriminate between typically developing children and children with cerebral palsy at group level. We additionally measured visibly lower muscular response strength in the pilot SDR group compared to the cerebral palsy group, reflecting the reduction in feedback activity due to the surgery. The group differences might have even been underestimated, for several reasons. First, the perturbation protocol used modeled MTU lengthening, instead of fascicle lengthening which is more directly related to stretch reflex responses [60]. Children with spastic cerebral palsy can experience limited fascicle lengthening—for example due to compliant tendons or increased baseline fascicle length prior to perturbations—and thereby low reflexive responses, despite high ΔMTV [60, 61]. This difference can arise due to increased activation in children with cerebral palsy [27], but also due to differences in tendon compliance [61, 62]. The error introduced by this factor can be analyzed by measuring muscle fascicle lengthening velocity (e.g., using ultrasound) instead of modeled MTV. Second, walking at an increased speed has been shown to result in increased reflex activity [22, 27, 63]. Children with cerebral palsy walked almost twice as slow in our study, but despite this had increased reflexes, as was also found in previous research [27]. Having children walk at similar speed might enlarge differences between groups [27], but it would be less feasible to increase walking speed for children with cerebral palsy and a less ecologically valid comparison to make typically developing children walk slower then their preferred walking speed [27, 28, 64]. Furthermore, we did not find any relation between walking speed and stretch hyperreflexia, as is explained in detail in Additional file 2. The final factor affecting differences between groups is that pathological gait patterns in cerebral palsy can change the mechanical responses. Different gait patterns can result in differences in initial ankle angles and relative fascicle length, which can influence the stimulation strength. We indeed found less ankle dorsiflexion in general in children with cerebral palsy, but this was corrected for in the muscular response strength. There were no apparent differences between outcomes caused by different gait patterns within the cerebral palsy group, as is presented in detail in the Additional file 3. However, these differences might appear when looking at more severely impaired gait. Further studies should look into the effect of more detailed but time-consuming analysis that includes correcting for initial fascicle length and relative ankle movement compared to the range of motion on the identification of individual’s identified muscle hyperreflexia. Despite these factors that could reduce the group effect, the protocol was still able to distinguish patients from typically developing children at group level and hence could be used in clinical settings to test treatment effects at group level.

**Fig. 5:**
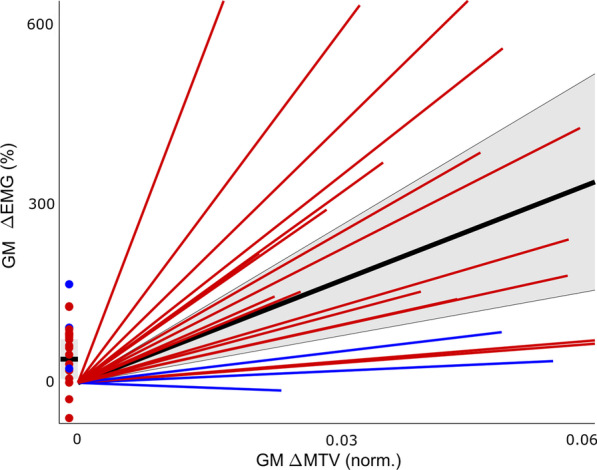
Between-participant variability in the muscular response for the GM. Lines represent the gain of the linear regression between musculo-tendon lengthening velocity and muscular responses, calculated for each participant individually from all measured strides from the perturbation trials (i.e., unperturbed and I1-I3). Dots represent the offset of the linear regression. Data is presented for typically developing children (bold black line represents mean, grey area represents mean ± standard deviation), children with cerebral palsy (red) and children who underwent SDR (blue). The offset of the linear regression is presented in a bar (typically developing) and circles (cerebral palsy and SDR), with higher values indicating high peak muscle activation levels at low levels of muscle lengthening velocity. The linear relation coefficient is presented as line steepness, with steeper lines indicating higher values of stretch hyperreflexia

The next important step towards clinical implementation is to validate the protocol for use in individual patients. While our experimental set-up was not aimed at this type of analysis, we did visualize the individual muscle responses of children with cerebral palsy. Figure 5 shows the large between-patient variability for the GM, as would be expected in such a heterogenous patient population. This variability cannot be explained by subject characteristics nor by perturbation characteristics—such as age, walking speed, musculo-tendon lengthening and relative increase in treadmill velocity—as is explained in more detail in Additional file 2. Although children with cerebral palsy had a higher muscular response strength on group level, some of these children had similar or even lower muscle response compared to typically developing children. This aligns with the recent notion that stretch hyperreflexia during passive movements is not strongly related to stretch hyperreflexia during active movements [17, 18]. Not necessarily all children with spastic cerebral palsy experience stretch hyperreflexia during gait, for instance due to a protective function of increased co-contraction [65, 66] or increased muscle stiffness [60]. Some researchers even suggest that stretch hyperreflexia does not negatively affect gait for patients with clinically diagnosed stretch hyperreflexia [66], although these findings are debated by other researchers [16, 28]. Our perturbation protocol can help provide insight into the contribution of stretch hyperreflexia to impaired gait. Our heterogenous results furthermore amplify the necessity of individual assessment of stretch hyperreflexia during functional movements. Treadmill perturbations can be a tool to evoke the stretch hyperreflexia and thereby explore if this is the primary cause of gait deviations on an individual basis.

### Recommendations

The applicability of this method for individual assessment should be further studied. Clinically relevant differences can be assessed by comparing stretch hyperreflexia evoked with perturbations pre- and post-treatments. Our results already indicate an effect of SDR surgery, which directly targets stretch hyperreflexia. This should be further studied by assessing more patients and including pre-SDR comparisons. The effect of other treatments, such as botulinum toxin injections, considered to affect spasticity, can also be studied using treadmill perturbations. This will add to the current clinical decision making and treatment evaluation, which mostly has to rely on less specific ordinal measures such as the Modified Ashworth Scale, and indirect functional measures such as passive ankle range of motion, walking speed, or—in more exceptional rehabilitation centers—the total muscle activity patterns during normal walking [67–69]. Larger sample sizes with more homogenous groups (e.g., only toe- or crouch walking) are needed to perform a generalizability study [70] to assess the smallest detectable differences of stretch hyperreflexia within and between patients.

This study assessed triceps surae muscles during gait, but the protocol could theoretically be redesigned to assess other muscles during functional activities, such as acceleration perturbations applied during late stance to assess rectus femoris hyperreflexia in early swing, or even during other activities such as hand biking to assess biceps hyperreflexia. It would be interesting to explore if stretch hyperreflexia expresses itself similarly in different functional activities, but we speculate a more similar expression during dynamic as opposed to passive tasks. While we show the feasibility of the protocol in more functional children, the applicability to more severe patients should be further explored. With some protocol adjustments, such as the usage of handrails on the treadmill to function as a surrogate hand-held mobility device and virtual feedback to reduce belt-cross stepping, the protocol might be well applicable across patients. Lastly, although this study focused on children with cerebral palsy, the protocol may well be applicable for use in patients with other central neurological system disorders, such as stroke and spinal cord injury, which should be further studied.

## Conclusion

In summary, we present a treadmill perturbation protocol to functionally assess stretch hyperreflexia in children with cerebral palsy. This study provides evidence supporting the feasibility, reliability and validity of the protocol. We provide a framework for future studies to analyze stretch hyperreflexia in patients with central nervous system disorders at an individual level for personalized interventions.

## Supplementary Information

**Additional file 1: Table S1.** Validity outcomes. Table contains values for the three different intensities (I1-3) for all construct validity parameters.

**Additional file 2**: Correlation analyses. This file contains correlation analyses between the level of stretch hyperreflexia and subject and perturbation characteristics.

**Additional file 3**: Deviating gait patterns. In this file we analyze if more impaired gait patterns affect the ability to evoke responses with the perturbations.

## Data Availability

The datasets used and/or analyzed during the current study are available from the corresponding author on reasonable request.

## Abbreviations

GM: Gastrocnemius medialis
MTL: Musculo-tendon lengths
MTV: Musculo-tendon stretch velocity
SDR: Selective dorsal rhizotomy
SO: Soleus
TA: Tibialis anterior
