# Supplementary material for: Functional assessment of stretch hyperreflexia in children with cerebral palsy using treadmill perturbations
Source: J Neuroeng Rehabil. 2021 Oct 18;18:151. doi: 10.1186/s12984-021-00940-1 (PMC8522046; doi:10.1186/s12984-021-00940-1)
Supplement: Supplementary file 1 — Additional file 1: Table S1. Validity outcomes. Table contains values for the three different intensities (I1-3) for all construct validity parameters. [file 12984_2021_940_MOESM1_ESM.pdf]

Supplementary Table 1: Validity outcomes

Parameters	I1 TD	I2 TD	I3 TD	Effect I TD	I1 CP	I2 CP	I3 CP	Effect I CP
**Kinematics**								
**Max Δangle (°)**								
Ankle	2,07 ± 0,85	3,65 ± 0,69	5,40 ± 1,34	**<0.001**	2,00 ± 0,83	3,05 ± 1,34	3,79 ± 1,40	**<0.001**
Knee	1,93 ± 1,08	2,75 ± 0,90	4,41 ± 1,64	**<0.001**	2,56 ± 1,17	3,78 ± 1,53	4,50 ± 1,90	**0.001**
**Max ΔMTL (norm.)**								
GM	1,12 ± 0,25	2,17 ± 0,31	2,99 ± 0,52	**<0.001**	0,87 ± 0,44	1,53 ± 0,73	2,15 ± 1,05	**<0.001**
SO	1,43 ± 0,59	2,47 ± 0,53	3,60 ± 0,89	**<0.001**	1,30 ± 0,56	1,95 ± 0,87	2,42 ± 1,02	**<0.001**
TA	-1,39 ± 0,56	-2,44 ± 0,51	-3,56 ± 0,81	**<0.001**	-1,30 ± 0,56	-1,98 ± 0,86	-2,49 ± 1,05	**<0.001**
**Max ΔMTV (norm.)**								
GM	13,30 ± 3,09	18,67 ± 2,68	25,04 ± 4,04	**<0.001**	13,12 ± 4,06	19,24 ± 3,68	23,05 ± 5,57	**<0.001**
SO	21,27 ± 4,44	28,91 ± 4,83	37,51 ± 7,40	**<0.001**	18,82 ± 7,03	25,31 ± 6,67	29,76 ± 9,78	**<0.001**
TA	-19,31 ± 3,59	-26,78 ± 3,65	-35,14 ± 5,36	**<0.001**	-17,84 ± 6,20	-24,27 ± 6,08	-28,72 ± 8,53	**<0.001**
**Max ΔEMG (%)**								
GM	114,35 ± 34,38	189,03 ± 48,60	230,92 ± 67,91	**<0.001**	147,88 ± 42,68	274,40 ± 131,02	359,53 ± 189,63	**<0.001**
SO	116,21 ± 32,12	196,90 ± 53,71	285,10 ± 160,90	**0.001**	142,93 ± 59,97	233,60 ± 109,65	282,69 ± 149,86	**<0.001**
TA	55,48 ± 16,72	62,98 ± 28,57	106,47 ± 39,83	**<0.001**	56,45 ± 32,49	135,43 ± 155,15	189,88 ± 204,63	**0.012**
**Duration (ms)**								
GM	123,86 ± 29,27	130,12 ± 21,32	139,29 ± 30,20	**0.026**	173,90 ± 50,86	235,61 ± 71,26	260,12 ± 85,92	**<0.001**
SO	117,00 ± 20,84	133,45 ± 16,72	146,71 ± 34,24	**0.001**	126,55 ± 33,69	175,03 ± 45,70	212,82 ± 60,79	**<0.001**
**Strength (norm.)**								
GM	11,65 ± 7,33	10,92 ± 2,97	9,95 ± 3,74	0.486	13,63 ± 5,66	14,75 ± 10,20	16,96 ± 8,81	**0.033**
SO	5,70 ± 2,84	7,22 ± 4,23	8,52 ± 5,65	0.062	10,76 ± 7,48	15,47 ± 17,37	11,10 ± 5,05	0.295
TA	-3,86 ± 3,63	-2,59 ± 1,18	-3,51 ± 1,64	0.622	-3,96 ± 4,25	-8,19 ± 8,65	-7,84 ± 7,56	0.051
**Co-contraction index**								
GM	0,48 ± 0,09	0,45 ± 0,08	0,45 ± 0,07	**0.008**	0,59 ± 0,12	0,57 ± 0,10	0,59 ± 0,12	0.975
SO	0,50 ± 0,08	0,46 ± 0,11	0,47 ± 0,07	**0.006**	0,63 ± 0,07	0,61 ± 0,08	0,60 ± 0,08	**0.018**

Values for the three different intensities (I1-3) are presented for all construct validity parameters. Abbreviations: with TD the typically developing group; CP the cerebral palsy group; P_{pert} the effect of perturbations (Helmert contrast); $P_{intensity}$ the effect of intensities (linear polynomial contrast,); P_{group} the difference between CP and TD; P_{inter} the interaction between CP and TD; MTL, musculo-tendon length; GM, gastrocnemius medialis muscle; SO, soleus muscle; TA, tibialis anterior muscle; MTV, musculo-tendon stretch velocity; CCI diff, difference in co-contraction index between unperturbed and highest intensity perturbations; Strength, muscular response strength. Mean ± standard deviations are presented. Significant p-values are expressed in bold.
