# Supplementary material for: Functional assessment of stretch hyperreflexia in children with cerebral palsy using treadmill perturbations
Source: J Neuroeng Rehabil. 2021 Oct 18;18:151. doi: 10.1186/s12984-021-00940-1 (PMC8522046; doi:10.1186/s12984-021-00940-1)
Supplement: Supplementary file 2 — Additional file 2: Correlation analyses. This file contains correlation analyses between the level of stretch hyperreflexia and subject and perturbation characteristics. [file 12984_2021_940_MOESM2_ESM.pdf]

Additional file 2: Correlation analyses

Given the heterogeneity in the group of participants with cerebral palsy, we explored the relationship between the reflex response and several parameters that might influence the response size. The reflex response was calculated similar to the gain in figure 5, as the linear relation coefficient between musculo-tendon lengthening velocity and muscular response, with higher values representing higher levels of stretch hyperreflexia.

First, we looked at subject characteristics including age, level of passive hyperreflexia as measured with the SPAT, gross motor function classification system (GMFCS) level, and baseline walking speed. Furthermore, the children with cerebral palsy had various different gait patterns, which can influence the effect of the perturbations. Therefore, we also assessed the relationship between reflex response and thee perturbation characteristics, being the maximum relative change in treadmill velocity (Peak Δv_{rel}) induced by the perturbation and the resulting increase in ankle dorsiflexion (Peak ΔAnkle angle) and maximum increase in musculo-tendon lengthening (Max ΔMTL) relative to baseline walking.

Scatterplots for all parameters are presented in Supplementary Fig. 1. Statistical analysis was performed for the cerebral palsy group and the typically developing group separately. The selective dorsal rhizotomy (SDR) group was left out of analyses, as the SDR surgery intervenes with normal and CP-pathological reflex responses. The relation between reflex response and age, walking speed, relative increases in treadmill velocity, induced ankle dorsiflexion, and induced musculo-tendon lengthening were explored using a Pearson correlation analysis. Outcomes of the Pearson correlation were squared to determine the explained variability on the hyperreflexia measure. Correlations for SPAT and GMFCS were calculated using Spearman's rho and for gender using partial eta squared.

None of the parameters were significantly related to the reflex response (See Supplementary Table 1). For the typically developing children, there appeared to be a trend for walking speed ($p=0.056$) and induced ankle dorsiflexion ($p=0.065$), but both correlations were weak ($r^2<0.2$ for both variables) and therefore cannot explain large parts of the variability in stretch hyperreflexia measures. Furthermore, this correlation was absent in the children with cerebral palsy ($p=0.487$ and $p=0.392$).

Supplementary Table 2: Correlations with stretch hyperreflexia

Parameters	Cerebral Palsy		Typically developing	
	r	p-values	r	p-values
Age	0.100	0.356	0.291	0.156
SPAT*	0.305*	0.250	-	-
GMFCS*	0.027*	0.921	-	-
Gender**	0.012	0.680	0.009	0.743
Walking speed	0.009	0.487	0.443	0.056
Peak Δv_{rel}	-0.081	0.383	-0.239	0.205
Peak ΔAnkle angle	0.074	0.392	-0.425	0.065
Max ΔMTL	0.006	0.491	-0.009	0.487

*A spearman's rho was calculated for these variables, given the ordinal nature of the parameters. ** Similarly, partial eta squared was calculated for gender, given the nominal nature of this parameter. Abbreviations: r = Pearson correlation coefficient; SPAT = passive spasticity assessment; GMFCS = gross motor function classification system; Δvrel = peak difference in treadmill velocity during the perturbation relative to participant's baseline belt velocity; ΔAnkle angle = increase in ankle dorsiflexion due to the perturbations relative to the baseline pattern; ΔMTL = difference in musculo-tendon length due to the perturbations relative to the baseline pattern.

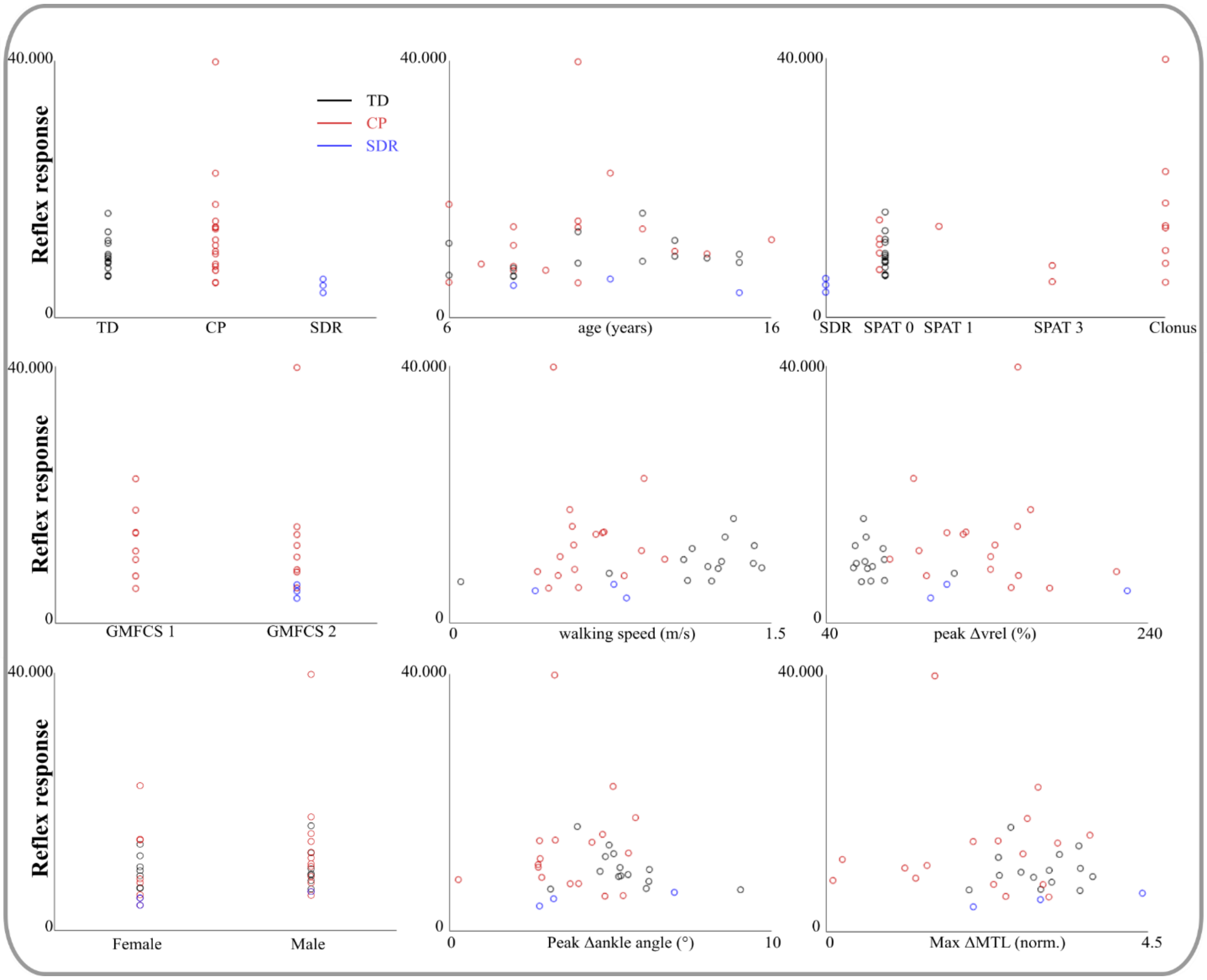

Supplementary Fig. 1. Scatter plots for hyperreflexia versus several parameters that potentially influence the hyperreflexia assessment. Reflex response reflects the increase in muscle activity relative to the increase in musculo-tendon lengthening velocity. Abbreviations: TD: typically developing children; CP = children with cerebral palsy; SDR = children with cerebral palsy who underwent selective dorsal rhizotomy surgery; SPAT = passive spasticity assessment. GMFCS = gross motor function classification system; Δvrel = peak difference in treadmill velocity during the perturbation relative to participant's baseline belt velocity; ΔAnkle angle = increase in ankle dorsiflexion due to the perturbations relative to the baseline pattern; ΔMTL = difference in musculo-tendon length due to the perturbations relative to the baseline pattern. Note that the SDR group was left out of the correlation analyses and only presented here for visual comparison.
