# Supplementary material for: Functional assessment of stretch hyperreflexia in children with cerebral palsy using treadmill perturbations
Source: J Neuroeng Rehabil. 2021 Oct 18;18:151. doi: 10.1186/s12984-021-00940-1 (PMC8522046; doi:10.1186/s12984-021-00940-1)
Supplement: Supplementary file 3 — Additional file 3: Deviating gait patterns. In this file we analyze if more impaired gait patterns affect the ability to evoke responses with the perturbations. [file 12984_2021_940_MOESM3_ESM.pdf]

Additional File 3: Deviating gait patterns

As more impaired gait patterns might affect the ability of treadmill perturbations to evoke ankle dorsiflexion, and thereby muscle lengthening and muscle responses, we more closely examined a selected group of cerebral palsy patients with most extreme deviations in the gait pattern by visual inspection of ankle and knee angles (see Supplementary Fig. 1). Selecting the participants with distinct excessive flexion during the stance phase resulted in three gait deviation groups: excessive knee flexion angle during stance (*n*=5), excessive ankle dorsiflexion during stance (*n*=5) and excessive plantar flexion during early stance (toe walking, *n*=2). We compared mechanical (peak Δankle dorsiflexion, Δknee flexion, and ΔMTV) and electrophysiological (ΔEMG for GM and SO) responses between these three subgroups and the remaining children with cerebral palsy (n=13). Statistical comparison was performed using unpaired rank-sum tests given the small group sizes.

The perturbation protocol seemed effective even in the more severely impaired gait patterns, as the mechanical and electrophysiological responses generally looked similar compared with the children with less abnormal gait patterns (Supplementary Fig. 1 D-I). Walking with excessive knee flexion did reduce the mechanical response in terms of peak ankle dorsiflexion (p=0.xx), possibly resulting in less SO MTV(p=0.16).

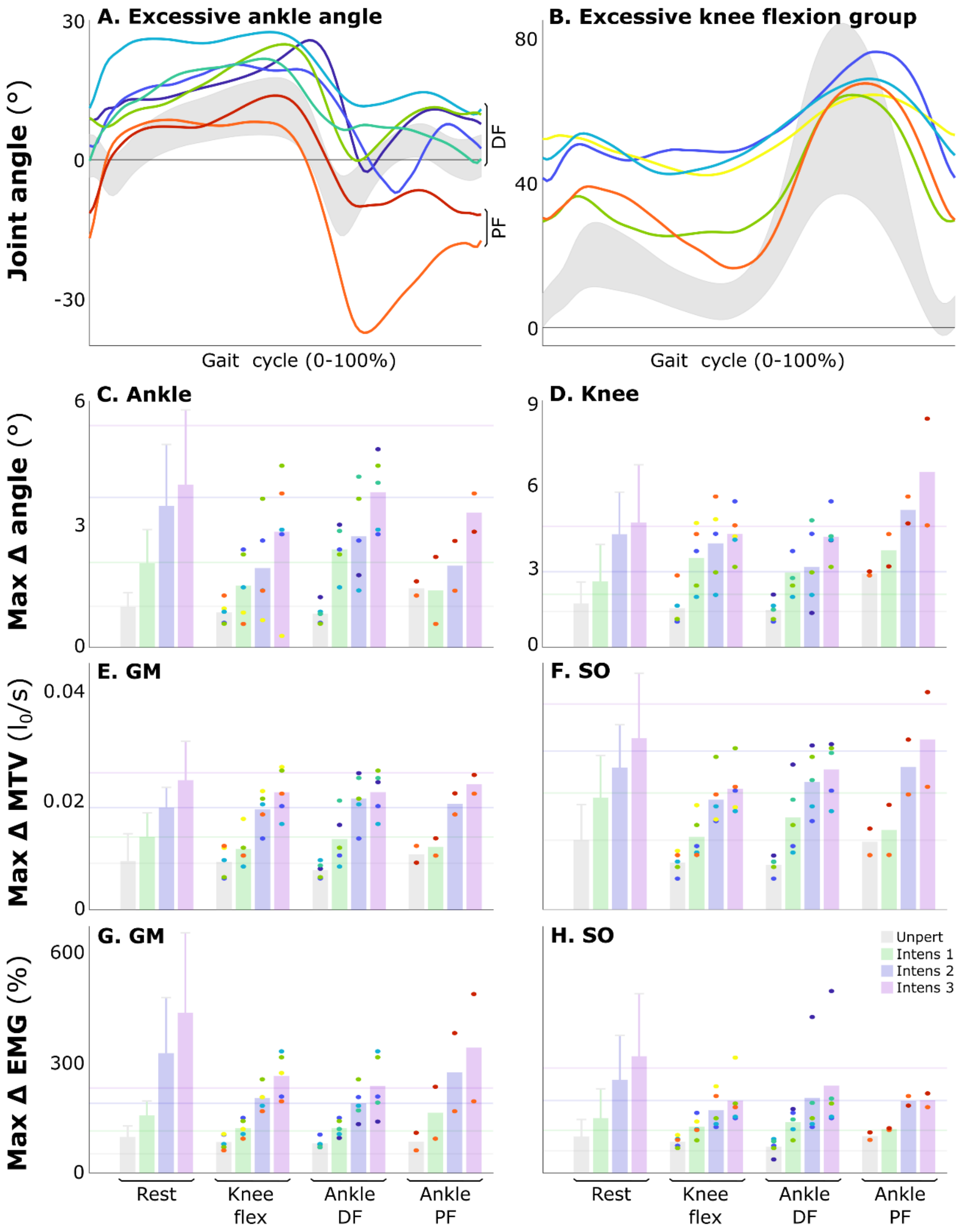

Supplementary Fig. 2. Effect of different gait patterns. **(A,B)** The time-normalized ankle and knee angle patterns for the participants of the different gait type groups: excessive ankle dorsiflexion (**B**; n=5), plantarflexion **(B)**; n=2) and knee flexion (**C**; n=5). Note that each participant has a unique color which is used throughout the figure, and three participants had both excessive ankle and knee angles. D-I) Bar graphs indicate mean and standard deviation for the average group (Rest) and individual average values are indicated for the deviating gait groups instead of error bars, due to the small sample sizes. Values are depicted for unperturbed steps (Unpert) and the three different intensities (Intens).
